# Development and validation of the patient-rated ulnar nerve evaluation

**DOI:** 10.1186/1471-2474-14-146

**Published:** 2013-04-26

**Authors:** Joy C MacDermid, Ruby Grewal

**Affiliations:** 1 Hand and Upper Limb Centre, 268 Grosvenor St, London, Ontario N6A 3A8, Canada; 2 School of Rehabilitation Science, McMaster University, Hamilton, Ontario L8S 1C7, Canada

## Abstract

**Background**

Compression neuropathy at the elbow causes substantial pain and disability. Clinical research on this disorder is hampered by the lack of a specific outcome measure for this problem. A patient-reported outcome measure, The Patient-Rated Ulnar Nerve Evaluation (PRUNE) was developed to assess pain, symptoms and functional disability in patients with ulnar nerve compression at the elbow.

**Methods**

An iterative process was used to develop and test items. Content validity was addressed using patient/expert interviews and review; linking of the scale items to International Classification of Functioning, Disability, and Health (ICF) codes; and cognitive coding of the items. Psychometric analysis of data collected from 89 patients was evaluated. Patients completed a longer version of the PRUNE at baseline. Item reduction was performed using statistical analyses and patient input to obtain the final 20 item version. Score distribution, reliability, exploratory factor analysis, correlational construct validity, discriminative known group construct validity, and responsiveness to change were evaluated.

**Results**

Content analysis indicated items were aligned with subscale concepts of pain and sensory/motor symptoms impairments; specific upper extremity-related tasks; and that the usual function subscale provided a broad view of self-care, household tasks, major life areas and recreation/ leisure. Four subscales were demonstrated by factor analysis (pain, sensory/motor symptoms impairments, specific activity limitations, and usual activity/role restrictions). The PRUNE and its subscales had high reliability coefficients (ICCs > 0.90; 0.98 for total score) and low absolute error. The minimal detectable change was 7.1 points. It was able to discriminate between clinically meaningful subgroups determined by an independent evaluation assessing work status, residual symptoms, motor recovery, sensory recovery and global improvement) p < 0.01. Responsiveness was excellent (SRM = 1.55).

**Conclusion**

The PRUNE is a brief, open-access, patient-reported outcome measure for patients with ulnar nerve compression that demonstrates strong measurement properties.

## Background

Compression of the ulnar nerve at the elbow (UNE), sometimes referred to as cubital tunnel syndrome, is the second most common compression upper extremity neuropathy [1]. The mean annual incidence has been estimated at 25 cases per 100,000 person-years, with the male to female difference being 33 to 17. Work-relatedness has been suggested; since males performing manual work have an elevated incidence of 57 cases/100,000 person-years [2]. A number of studies have related development of symptoms to occupational activities including sustainable flexion postures [3,4] and repetitive elbow movement; or sporting activities like cycling [5]. The prevalence of UNE is 3.5 times higher in people who report occupational activities that involve ‘holding a tool in position’ [4] compared to workers in the same setting who do not perform this task. However, other physical and psychosocial factors can also contribute to UNE [6]. In a multidimensional risk study, smoking, education level and work experience were identified as risk factors; whereas, gender, BMI, alcohol consumption, trauma to the elbow, diabetes mellitus, and hypertension were not [7].

Evidence on the management of UNE is problematic; and indicates a need for better outcome reporting. A Cochrane review was only able to locate six low-quality clinical trials relating to management of ulnar neuropathy. This review concluded “ *available evidence is not sufficient to identify the best treatment for idiopathic ulnar neuropathy at the elbow on the basis of clinical*, *neurophysiological and imaging characteristics*. *We do not know when to treat a patient conservatively or surgically*.” [8] This review recommended that future research would be improved by the use of validated disease-specific clinical outcome measures. Our systematic review of prognosis following anterior ulnar nerve transposition faced similar challenges [9]. Although, we were able to locate 26 studies addressing prognosis following ulnar nerve transposition surgery, only two of these were high-quality. We found profound inconsistencies in the design and conclusions of available studies; and were unable to make any conclusions based on the studies located. We found the lack of standardized evaluation of outcomes was a substantial barrier to the conduct of an effective systematic review.

Although the evidence about treatment and prognosis has flaws, it is clear that ulnar nerve compromise can lead to substantial disability. In a qualitative study of patients with ulnar nerve palsy, the majority of people had difficulties with simple, everyday tasks including holding soap,eating, buttoning clothes, holding a glass or lifting small objects [10]. Ulnar nerve compression causes less compromise to the ulnar nerve, but also results in substantial hand impairments [11]. Despite the unique and potentially profound consequences of ulnar nerve problems, there has been little attention to developing and validating UNE disease-specific patient report outcome measures. A systematic review of the outcomes measures used to assess outcomes of UNE identified 42 clinical studies [12] that used 21 different health outcomes measures including 2 generic instruments; 10 nonstandardized measures; 3 symptom-specific patient-reported instruments; and 6 patient questionnaires. A review of standardized rating systems for evaluation of the elbow did not locate any tools specific for UNE [13]. Further, this review noted that most scoring systems used to evaluate elbow function have limited supporting psychometric data.

In 2006, Mondelli and colleagues described a 9-item UNE scale developed in Italian and then translated to English [14]. It was developed in Italian, and published with an English translation. Data from this questionnaire were compared to nerve physiological features, clinical measures and the Boston Carpal Tunnel Questionnaire [15]. The scale had low correlation to electrophysiology grade, and moderate correlation to clinical severity. Test–retest reliability in the first 44 patients was excellent (0.97) and responsiveness was acceptable in 25 patients followed 6–8 months of conservative management (effect size = 0.46). The item development/reduction process and translation process were not reported. It has not been widely used in subsequent research.

The purposes of this paper are to report the development and validation of a patient-report outcome measure that is designed for use in patients with ulnar nerve compression. Specific objectives include to describe the development process, reliability, content and construct validity, and factor/structural validity.

## Methods

The Patient-Rated Ulnar Nerve Evaluation (PRUNE) was developed based on iterative revisions and stakeholder consultation. A formal structured examination of content validity; a statistical analysis of test-retest reliability, factor structure, and construct validity were used for item reduction and evaluation of the final instrument.

### Scale development process

The first author has developed previous PRO that have a common structure of pain, specific activity and usual activity subscales [16-19] disorders. This structure informed the structure of the PRUNE. The specific items were developed through patient interviews, epidemiological and biomechanical studies. Symptoms that were relevant to patients with ulnar nerve compression neuropathy were grouped into: pain and ulnar-nerve “specific” sensory symptoms or motor symptoms. Together with the specific activities and usual functional activities, 4 subscales were derived. A long version of the instrument was completed by all participants and the final reduced scale was determined by a structured item reduction process. Cross correlations between items, factor analysis, inspection for floor and ceiling effects and item distribution were used to reduce the item pool to the optimal set of items.

### The patient-rated ulnar nerve evaluation (PRUNE)

**Figure 1:**
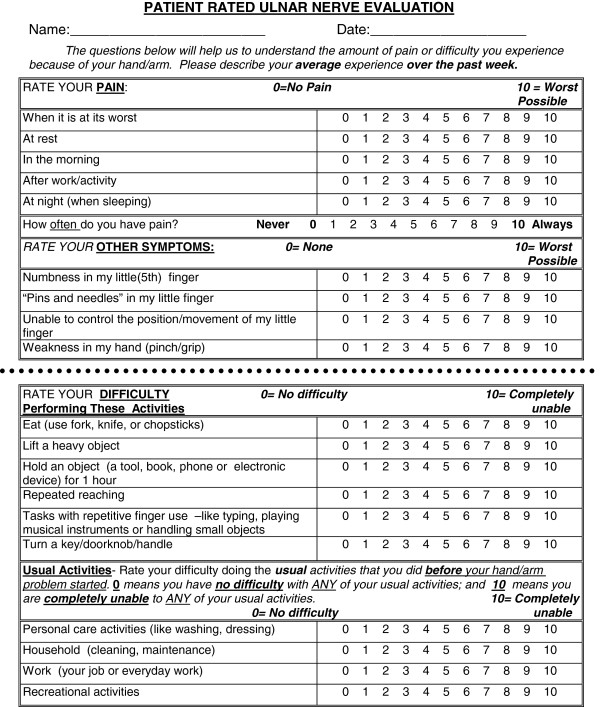
Patient-rated ulnar nerve evaluation.

The final version of the PRUNE is presented in Figure 1. The PRUNE is a 20-item scale that measures pain, sensory/motor symptoms and functional disability in patients with UNE. The 20 items include: 6 pain, 4 sensory/motor symptoms 6 specific activity; and 4 usual activity (personal care, household, work and recreation) items. Each item is scored on a scale from zero (none/no difficulty) to 10 (worst possible/completely unable). Each subscale is scored by adding the component questions (pain/60, sensory/motor symptoms/40, specific activity/60, usual activity/40). The total score is calculated to range from 0–100 points with zero meaning no symptoms or difficulty and 100 being worst possible symptoms and completely unable to do all functional activity. The total score equally weights the 10 items on symptoms and 10 functional items (by dividing the grand total by 2).

The individual items retained during item reduction are in the appended instrument (Figure 1) which is the final format for the PRUNE. Items modified in the final stage of beta testing and the rationale is listed in Table 1 to document the rationale for item reduction.

**Table 1:** Patient demographics

**Characteristic (n = 89)**	**Descriptors**
Age in years	Range 20-81
	Mean = 52.75; SD- 12.86
**Gender**	%
Male	75
Female	25
**Workers compensation status**	%
No	82%
Yes	15%
Claim in progress	3%
**Type of work**	%
Heavy repetitive	37
Heavy intermittent	18
Light repetitive	18
Light intermittent	28
**Work status**	%
Normal duties	21
Light duties at same work place	15
Off work due to arm	18
**Handedness**	%
Left	7
Right	93
**Surgical side**	%
Left	58
Right	43
**Electrodiagnostic findings**	37.47 m/s
AE-MNCV	50.65 m/s
BE-MNCV	13.17 m/s
DNCV (m/s)	3.79 mV
AE-CMAP	17.29%
BE-CMAP	4.98 mV
CMAP ADQ amplitude	5.74 μV
SNAP digit V	21.42 ms
SNCV digit V (ms)	75%
Surface EMG confirmed	89%
Needle EMG confirmed	
**McGowan stage [1]**	%
0	0
I	13
II	46.4
III	40.6

*CMAP*, Compound motor action potential. *SNAP*, sensory nerve action potential. *MNCV*, Motor nerve conduction velocity. AE-CMAP CMAP from above elbow to below elbow. AE-MNCV MNCV from above elbow to below elbow. BE-CMAP CMAP from below elbow to wrist. BE-MNCV MNCV from below elbow to wrist. Reference List [1] McGowan AJ: **The Results of Transposition of the Ulnar Nerve for Traumatic Ulnar Neuritis.***J Bone Joint Surg* 1950, **32:** 293–301.

### Treatment

This study was not designed to study intervention effects. However, response to treatment was used as a context to evaluate the clinical measurement property of responsiveness. Patients underwent a submuscular or subcutaneous ulnar nerve transposition using established procedures [20-22].

### Comparison study measures

#### The SF-36

The SF-36 is a 36-item scale that addresses general health. Subscales address Physical Function, Physical Role, Bodily Pain, General Health, Vitality, Social Function Emotional Role, and Mental Health. These subscales are summarized into Physical and Mental Summary Component scores. While the SF-36 is less responsive than disease-specific scales [23,24], it is a valid indicator of general health status in musculoskeletal disorders [25]. General health status measures are commonly used in construct validation and are expected to have a low to moderate relationship with a disease-specific measure like the PRUNE.

#### The bishop scale

The Bishop Scale (sometimes referred to as Kleinman and Bishop) [26] is a clinician administered measure developed specifically for UNE. The scale addresses: satisfaction, improvement, severity of symptoms, work status, leisure activity, strength, and sensibility. There is no description of standardized application of the tool; nor has reliability, validity or responsiveness been reported [12]. The Bishop scale was administered by an independent evaluator and the items were used for criteria to test known group validity since the scale provides criteria for a number of clinically relevant subgroups.

#### Patient recruitment

Patients (n = 89) were diagnosed in a multi-stage process. The preliminary diagnosis was made by the family physician who then referred the patient for electrodiagnosis and examination by a physical medicine physician. The electrodiagnostic parameters as reported in patient demographics and clinical presentation were considered to make the definitive diagnosis. Patient with a confirmed ulnar neuropathy were sent to surgical consultation with a fellowship trained hand surgeon, who again confirmed the diagnosis again using electrodiagnostic findings and clinical examination. Patients undergoing anterior nerve transposition were approached and agreed to participate in this study. Inclusion criteria included electrodiagnostically confirmed ulnar nerve compression at the elbow, persistent symptoms for at least 3 months with failed conservative management, and able to return for follow-up. Exclusion criteria included: unable to complete self-report forms, central or spinal neurological disorders, other neuropathy affecting the hand (excluded by electrodiagnosis) and medical conditions that precluded participation. Respondents completed the full version of the PRUNE within 2 weeks prior to surgery; and again 3 and 24 months following surgery.

The study was approved by the Western University Ethics Board. Written informed consent for participation in the study was obtained from all participants; none were under 18 years of age.

### Analyses

#### Scale distributions and floor/ceiling

Box plots were used to examine the distribution of scores for individual items and subscales to examine potential floor/ceiling effects or distribution problems.

#### Content validity

Content validity is fundamental to scale validity and was assessed by four methods. Patient interviews were used during development and reduction of items, pilot testing and the psychometric study to assess content relevancy. The prototype instrument was reviewed by patients, 3 physical therapists, 1 physiatrist, 3 orthopedic surgeons and 2 research assistants. Patients and experts provided feedback on the appropriateness and wording of the items.

Structured content analysis was performed using 2 methods. The International Classification of Functioning Disability and Health (ICF) linking procedures were performed according to established linking rules [27,28]. ICF coding provides a common international language to describe the elements of body structure, function, disability and environment contained in questionnaire items. The Item Perspective Classification was used to perform a 2-level classification of type of decision (rational/emotional) and content of items (psychological, social, biological, inorganic or pure experience). More detail on this coding method can be found at https://sites.google.com/site/ipcframework/.

#### Reliability

A subset of patients was re-tested 2–7 days after their completing the PRUNE. The following statistics were calculated to establish the reliability of the PRUNE:

a) reliability intraclass correlation coefficients (2,1) [29],

b) Standard error of measurement:

$$\mathrm{SEM}=\mathrm{standard}\;\mathrm{deviation}\;\times \sqrt{1-\mathrm{reliability}\;\mathrm{coefficient}}$$

and

c) Minimal detectable change (90% confidence) [30,31]

$${\mathrm{MDC}}_{90}=\mathrm{SEM}\;\times \;\mathrm{z-value}\;\mathrm{of}\;1.64\;\times \;\sqrt{2}$$

#### Structural validity

Exploratory factor analyses (principal components analysis using varimax rotation) were used to assess how scale items distributed into subscales [32]. Analysis was performed on data collected at baseline which included a larger subset of items; and these results contributed to decisions about item reduction. Thus, the factor analysis performed at 3 and 24 months included only the final items.

#### Construct validity

The following hypotheses/expectations were constructed to assess construct validity of the PRUNE based on convergent relationships expected from theoretical and evidence perspectives [33,34]. The strength of these associations was assessed using Pearson correlations.

1. Scales measuring the same construct (pain or functional disability) should demonstrate high correlation (indicated by r >0.75)

2. Pain and function should moderately correlate (0.40-0.75)

3. General health status would demonstrate low to moderate correlation with PRUNE subscale and total scores. PRUNE scores should correlate more highly with physical function than with mental function. Pain was expected to correlate with both physical and mental health status.

#### Constructed hypotheses for known-group validity

The following known group differences were tested (by ANOVA) to assess construct validity of the PRUNE. The subgroups were defined by an independent assessor through patient interview and examination using criteria defined by Kleinman and Bishop [26].

1. Patients who perceived their global rating of change at 2 years as: improved, versus no change, or worse

2. Patient who were asymptomatic versus those who had mild-occasional, moderate, or severe symptoms

3. Patients who were able to return to work at their regular job versus those who are unable to work because of continued symptoms

4. Patients whose leisure was unlimited versus those who were limited

5. Patients who had both grip and pinch 80% of opposite hand versus those who had either grasp or pinch reach 80% of opposite hand versus those both where grip and pinch less than 80% of opposite hand

6. Patients who had normal sensibility defined as two-point discrimination less than 5 mm versus those where it was abnormal greater than 5 mm.

#### Responsiveness

Changes over time were evaluated by calculating a standardized response mean (change score divided by the standard deviation of the change scores) and effects size (change score divided by the standard deviation of the initial scores) [35].

## Results

The final version is presented in Figure 1. Patients (See Table 1 for demographics) completed the PRUNE with few missing items (<1.0%).

### Content validity

A prototype 25-item scale was developed based on items obtained item generation and early refinement procedures (expert and patient feedback). Subsequent item reduction of the 25-item prototype scale was based on statistical analyses and cognitive interviewing [36,37] Cognitive interviewing with patients indicated wide variability in interpretation (and use) in relation to the telephone item that led to modification of that item (Table 2).

**Table 2:** Items that did not meet inclusion in the final instrument version of the PRUNE

**Item**	**Rationale for exclusion/modification**
Deleted items	
Pain during activity	Contamination of pain and function concepts within item
Combing hair	Not appropriate to all respondents; higher level of missingness
Using arms rise from a chair	Highly correlated to tying shoes; less responsive
Pulling a heavy object	Correlated to lifting a heavy object; lifting a 10 kg weight
Lifting a 10 kg weight	As above; not all respondents understand 10 kg
Putting on a coat	Respondent feedback suggested less relevant; poor item performance overall
Doing up buttons	Cognitive interviews and task analysis suggests this item does not reflect sensory impairment of the ulnar nerve— the concept of fine motor control was covered by finger use question
Modified items	
At night (while sleeping)	Shift workers were not sure if at night meant during sleep or their night activity which was work. Added ( while sleeping to clarify)
Hold an object	Item performance variable; however strong bio mechanical support and patient endorsement that some type of holding object with arm bent was difficult. Qualitative interviews indicated that respondents used a variety of reading devices and positions; and were not always clear that it meant a continuous activity. Item modified to specify one hour interval and allow multiple options for the object that was held clarifying that the elbow is bent
Eating	Added specification of different eating utensils for cultural transferability
Control of the small finger	Different respondents use either small, little or fifth finger to indicate the fifth digit. Motor dysfunction related to the ulnar comprise could include either deformity, lack of motor control— lay terms were used for these phenomena.
Finger use	Finger use was a common difficulty reported by patients. It was most remarkably noted for keyboarding or musical instrument use but not all respondents perform these tasks therefore the question was modified to: Repeated finger movement (like when typing, playing instruments or moving small objects )

After item generation and initial iterative changes to the PRUNE, a larger potential instrument was tested on respondents. This larger subset of items underwent both cognitive interviewing and statistical analyses to determine the final subset of items included in the PRUNE.

ICF codes for the items are presented in Table 3. Pain items were coded to the ICF code for “Sensation of unpleasant feeling indicating potential or actual damage to some body structure felt in either one or both upper limbs, including hands (b28014). Concepts that relate to severity like frequency and intensity are not linked to ICF. Experts considered pain assessment fundamental to self-report and approved the range of qualifiers used for the pain items. The sensory motor subscale captures four separate ICF codes addressing touch function, sensation related to the skin; control of movement and muscle power. The Specific Activities Subscale was linked to six ICF disability codes at the third or fourth level and comprises codes that describe specific ADLs. The Usual Activity items coded to a high ICF level, i.e., chapter is consistent with the intent of this subscale to address broad domains of usual activity. In ICF language this subscale addresses self-care, household tasks, major life areas and recreation and leisure.

**Table 3:** ICF codes for patient-rated ulnar nerve evaluation items

**Pain items**	**ICF**	**Meaning in ICF language**
**Code(s)**		
When it is at its worst	b28014 - Pain in upper limb	Sensation of unpleasant feeling indicating potential or actual damage to some body structure felt in either one or both upper limbs, including hands.
At rest	b28014 - Pain in upper limb	
In the morning; #	b28014 - Pain in upper limb	Note: The descriptors of after work or while sleeping refer to a time point NOT an activity so they are not coded in ICF
After work/activity#	b28014 - Pain in upper limb	
At night#	b28014 - Pain in upper limb	
How **often** do you have pain#	b28014 - Pain in upper limb	
**Other symptom items:**		
Numbness in my little finger	b265 - Touch function	Sensory functions of sensing surfaces and their texture or quality. Inclusions: functions of touching, feeling of touch; impairments such as numbness, anaesthesia, tingling, paraesthesia and hyperaesthesia
“Pins and needles” in my little finger	b265 or b280 - Sensation related to the skin	Sensations related to the skin such as itching, burning sensation and tingling. Inclusions: impairments such as pins and needles sensation and crawling sensation
Cramping or unable to control my little finger	b760 - Control of voluntary movement functions	Functions associated with control over and coordination of voluntary movements. Inclusions: functions of control of simple voluntary movements and of complex voluntary movements, coordination of voluntary movements…
Weakness in my hand (pinch/grip)	b7300 - Muscle power functions	Functions related to the force generated by the contraction of a muscle or muscle groups. Inclusions: functions associated with the power of specific muscles and muscle groups, muscles of one limb, one side of the body, the lower half of the body, all limbs, the trunk and the body as a whole; impairments such as weakness of small muscles in feet and hands…
**Specific activities items**		
Eat (use fork, knife, or chopsticks)	d550 - Eating	Carrying out the coordinated tasks and actions of eating food that has been served, bringing it to the mouth and consuming it in culturally acceptable ways, cutting or breaking food into pieces, opening bottles and cans, using eating implements, having meals, feasting or dining.
Lift a heavy object	d4300 - Lifting	Raising up an object in order to move it from a lower to a higher level, such as when lifting a glass from the table.
Hold an object with my elbow bent (a telephone, tool, book, phone or electronic device)	d4401- Grasping	Using one or both hands to seize and hold something, such as when grasping a tool or a door knob
Repeated reaching	d4452 - Reaching	Using the hands and arms to extend outwards and touch and grasp something, such as when reaching across a table or desk for a book
Tasks with repetitive finger use –like typing, playing musical instruments or handling small objects	d440 - Fine hand use	Performing the coordinated actions of handling objects, picking up, manipulating and releasing them using one’s hand, fingers and thumb, such as required to lift coins off a table or turn a dial or knob. Inclusions: picking up, grasping, manipulating and releasing Exclusion: lifting and carrying objects (d430)
Turn a key/doorknob/handle	d4453 - Turning or twisting the hands or arms	Using fingers, hands and arms to rotate, turn or bend an object, such as is required to use tools or utensils
**Usual activities items**		
Personal care activities (like washing, dressing)	D5 - Chapter 5 Self-Care	Caring for oneself, washing and drying oneself, caring for one’s body and body parts, dressing, eating and drinking, and looking after one’s health
Household (cleaning, maintenance)	D630-649 - Household tasks	Range of tasks within domestic life that pertain to household but no specific group definition
Work (your job or everyday work)	D8 - Chapter 8 Major Life Areas	Carrying out the tasks and actions required to engage in education, work and employment and to conduct economic transactions.
Recreational activities	D920 - Recreation and leisure	Engaging in any form of play, recreational or leisure activity, such as informal or organized play and sports, programmes of physical fitness, relaxation, amusement or diversion, going to art galleries, museums, cinemas or theatres; engaging in crafts or hobbies, reading for enjoyment, playing musical instruments; sightseeing, tourism and travelling for pleasure

Using Item Perspective Classification all items were rational judgments because patients needed to recall information over the past week. Pain, symptoms and specific activities were classified as rational biological judgments. The first item of the usual activities subscale addresses personal care and hence falls under a rational judgment within the biological domain. The remaining three items (household, work and recreation) were classified as rational judgments about the social domain.

### Item behavior/distributions

**Figure 2:**
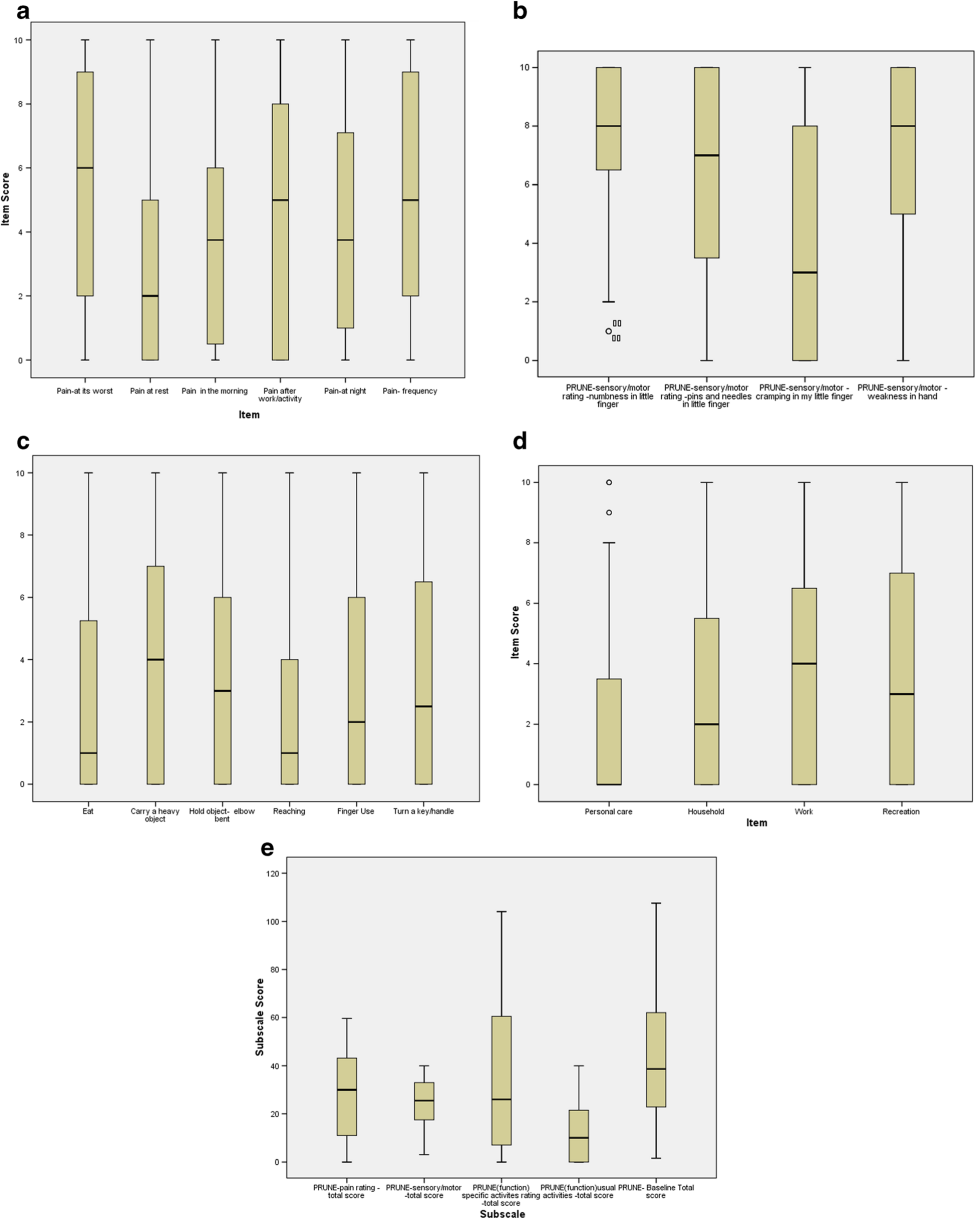
**Boxplots of subscale items at baseline. a:** Boxplot of Pain Items. **b:** Boxplot of Sensory/motor Symptoms Subscale. **c**: Boxplot of Items in Specific Activity Subscale. **d**: Boxplots of Items in Usual Activity Subscale. **e**: Boxplot of Subscale and Total Scores of Baseline PRUNE.

The full range of scores was used for all items except for numbness. The boxplots of items for each subscale at baseline (Figure 2 a, b, c, d and e).

The 6 pain items (Figure 2a) indicate that least and most pain items behaved as expected; with the remainder of items falling in a moderate range. From the sensory/motor symptoms subscale (labeled “Other Symptoms), numbness, pins and needles and weakness in the hand had high overall ratings; whereas, the motor control item was less severely affected. Numbness was the only item not endorsed by some individuals. Item responses for baseline specific functional activities (Figure 2b) indicated that “Carrying a heavy object” and holding an object with the arm bent were difficult tasks; whereas, eating and use of the telephone had lower ratings. Work was the most difficult “usual activity”.

The subscale and total scores distributions and indicators of central tendency are reported in boxplots 2e. These illustrate that subscales are normally distributed.

### Reliability

High ICCs were obtained with all subscales exceeding 0.90 except for usual activities (0.87). The ICC was 0.98 for the total score. Lower limits of the confidence interval were high with the exception of usual activities where the confidence interval was wide (Table 4). The standardized response mean (SEM) and Minimal Detectable Change (MDC) were 3.1 and 7.2 respectively for the total score (Table 4).

**Table 4:** Reliability of PRUNE subscales and total scores

**Subscale**	**Mean score test**	**Test SD**	**Mean score retest**	**Re-test SD**	**ICC**	**95% CI**	**Standard error of measurement (SEM)**	**SEM%**	**Minimum detectable change (MDC)**	**MDC%**
Pain/60	25.1	17.3	25.0	18.0	0.98	0.91-0.99	2.5	4	5.8	10
Sensori-motor symptoms/40	12.6	7.9	14.0	9.8	0.91	0.70-0.98	2.7	7	6.2	15
Specific activities/40	35.7	31.9	36.0	30.5	0.99	0.97- 0.999	3.1	8	7.3	18
Usual activities/40	10.8	13.2	13.4	13.0	0.87	0.57-96	4.7	11	11.0	27
Total Score/100	34.4	21.5	36.0	23.1	0.98	0.95-0.997	3.1	3	7.2	7

*SD*, Standard deviation; *ICC*, Intraclass correlation coefficient; *CI*, Confidence interval. A random sample of patients and visits were selected to do a retest questionnaire in 2–7 days. The % of SEM and MDC as a proportion of scale allows for comparability of these across different sized subscales.

### Construct validity

**Figure 3:**
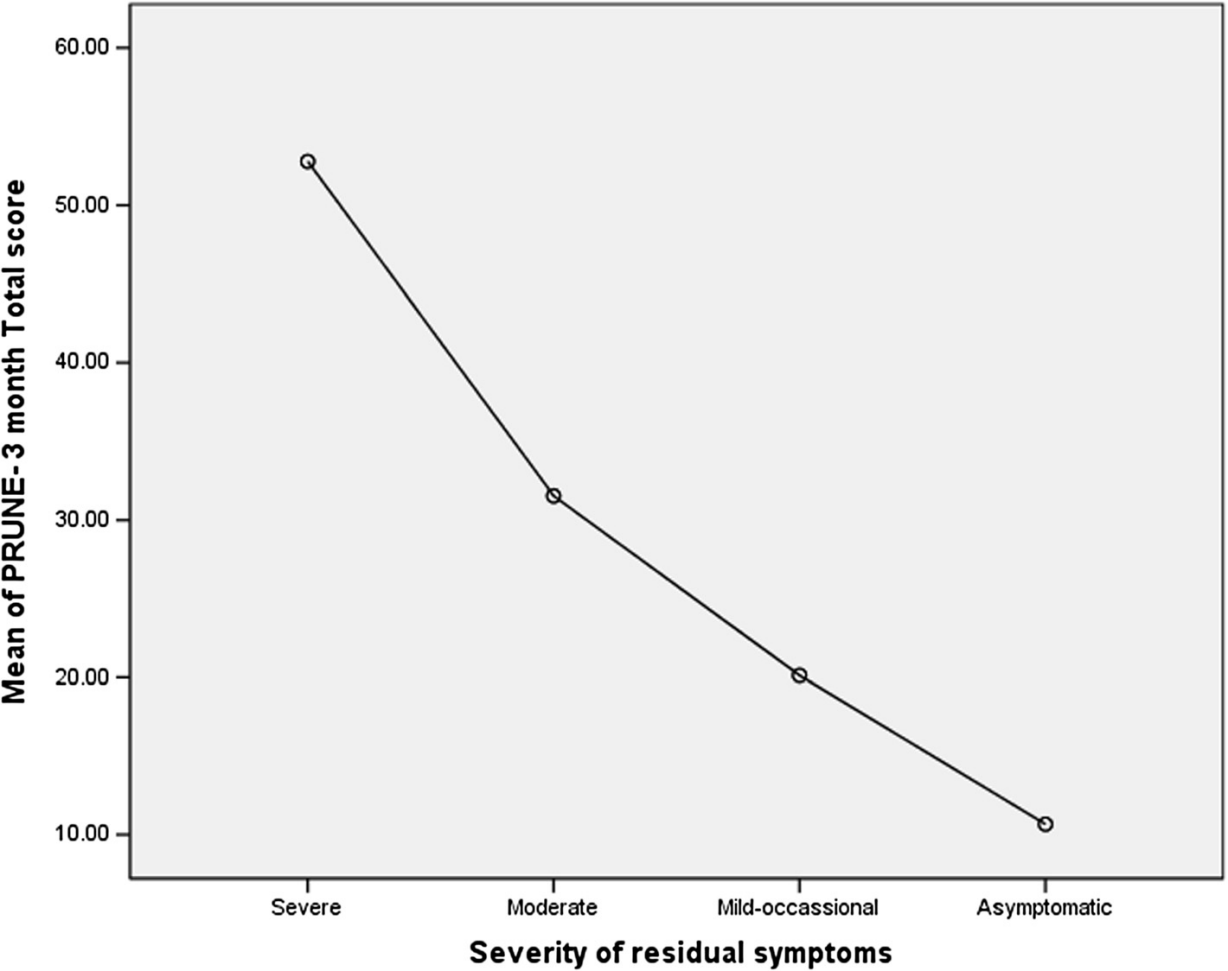
PRUNE symptom score at 3 months post-op.

The PRUNE was highly discriminative between different functional outcomes across all of the defined subgroups (See Tables 5 and 6). The follow-up scores were significantly different between subgroups based on whether they were working or not (16.5 vs. 53.8), able to do their normal activities or not (10.8 vs. 37.8) or had 2-point discrimination < 5 mm or not (21.9 vs. 26.8). People who reported their scores were the same or worse after surgery had PRUNE scores over 40 at follow-up; whereas, those who reporting being better had a score of 14.1 (see Tables 5 and 6 for subgroup scores). There was a linear trend to PRUNE scores based on whether patients reported mild to severe disability (Figure 3).

**Table 5:** Construct validity indicated by binary clinical subgroups at 24 months

**Clinically meaningful subgrouping**	**Subscale**	**Scores**			
**Work status**		**Not working because of ulnar neuropathy (n = 9)**		**Working or able to work at previous job (n = 34)**	
		Mean	SD	Mean	SD
	Total score	53.8	14.6	16.5	16.2
	Pain	34.1	16.1	11.2	12.5
	Symptoms	27.8	12.0	11.6	10.7
	Specific activities	48.4	12.7	13.0	16.9
	Usual activities	19.9	7.0	5.4	8.7
**Ability to do activities**		**Limited (n = 25)**		**Not limited (n-27)**	
		Mean	SD	Mean	SD
	Total score	37.8	18.0	10.8	12.0
	Pain	23.0	16.7	8.8	8.9
	Symptoms	20.6	11.6	8.1	9.1
	Specific activities	34.5	22.7	4.9	13.3
	Usual activities	15.0	8.3	1.7	2.5
**Sensibility**		**Abnormal (> 5 mm) (n-14)**		**Normal (n = 28)**	
		Mean	SD	Mean	SD
	Total score	26.8	24.2	21.9	21.4
	Pain	16.3	17.9	18.4	15.3
	Symptoms	22.0	12.5	10.9	11.8
	Specific activities	25.5	27.0	15.4	19.3
	Usual activities	10.1	10.6	7.1	9.3

All subscale scores and the total score were significantly different between subgroups for both work status and activity status. For sensory subgroups only the sensory/motor (SM) symptoms scale was different between subgroups. All p-values were significant at p < 0.01.

**Table 6:** Differences between clinically meaningful subgroups

**Clinically meaningful subgroups**	** Subscale**	**Scores**							
**Overall change following surgery**		**Worse (n = 6)**		**Unchanged (n = 9)**				**Better (n = 45)**	
		Mean	SD	Mean		SD		Mean	SD
	Total score	46.7	19.3	43.9		22.2		14.1	17.1
	Pain	28.6	22.6	20.1		17.0		9.9	12.9
	Symptoms	27.1	13.3	27.1		11.0		8.9	9.9
	Specific activities	40.8	28.8	35.6		24.8		10.9	18.8
	Usual activities	16.8	4.3	12.6		11.0		5.4	9.4
**Hand strength**		**Both grip and pinch < 80% of opposite hand (n = 8)**		**Either grip or pinch < 80% of opposite hand (n = 7)**				**Grip and pinch both > 80% of opposite hand (n = 25)**	
		Mean	SD	Mean		SD		Mean	SD
	Total score	33.1	18.6	22.1		17.9		14.8	16.7
	Pain	20.8	16.4	16.0		12.9		13.4	9.4
	Symptoms	19.0	14.1	13.5		9.4		9.8	9.5
	Specific activities	24.8	39.7	20.7		16.0		11.2	18.2
	Usual activities	13.2	11.1	9.0		9.0		5.3	7.6
**Residual symptoms**		**Severe**		**Moderates**		**Mild occasional**		**Asymptomatic**	
		Mean	SD	Mean	SD	Mean	SD	Mean	SD
	Total score	61.1	29.0	40.4	17.7	17.3	14.5	6.6	16.0
	Pain	39.6	24.7	26.9	15.3	10.7	10.7	4.8	10.7
	Symptoms	33.8	9.3	23.3	10.8	11.7	9.5	4.3	11.4
	Specific activities	54.4	3.4	34.0	25.0	11.7	16.0	5.0	2.6
	Usual activities	19.0	9.9	18.2	9.4	4.6	6.8	1.1	2.7

Although there were variations between subsets of scores; there was a significant linear trend across all scores; patients in the best clinical outcome category always were significantly better than the lowest clinical outcome category regardless of the subscale (p < 0.01).

Correlations coefficients were more strongly associated with the physical health domains on the SF-36 in comparison to the mental health domains (Table 7). The sensory/motor symptoms subscale correlated most strongly with overall physical health status indicating the importance of the ulnar nerve symptom items. Stronger correlations were observed between more conceptually similar subscales of the PRUNE and SF-36. All of these findings supported the construct validity.

**Table 7:** Construct validity indicated by PRUNE scores to general health status subscales

	**PRUNE score (2-year)**				
**SF- 36 subscale**	**Pain**	**SM symptoms**	**Specific activities**	**Usual activity**	**Total score**
	76	76	75	75	76
SF36 - 2-Physical Functioning Subscale	−0.5^**^	−0.46^**^	−0.39^**^	−0.36^**^	−0.52^**^
SF36 - 2 -Role -Physical Subscale	−0.35^**^	−0.41^**^	−0.16	0.08	−0.25
SF36 - 2-Bodily Pain Subscale	−0.59^**^	−0.68^**^	−0.53^**^	−0.35^**^	−0.64^**^
SF36- 2 -General Health Subscale	−0.33^*^	−0.34^**^	−0.19	−0.25	−0.33^*^
SF36 - 2-Vitality Subscale	−0.48^**^	−0.37^**^	−0.21	−0.12	−0.36^**^
SF36 - 2 -Social Functioning Subscale	−0.60^**^	−0.45^**^	−0.31^*^	−0.09	−0.45^**^
SF36 - 2 -Role- Emotional Subscale	−0.39^**^	−0.41^**^	−0.30^*^	−0.16	−0.38^**^
SF36 - 2 -Mental Health Subscale	−0.34^**^	−0.19	−0.20	−0.07	−0.25
SF36 - 2 -Physical Component Summary	−0.58^**^	−0.68^**^	−0.42^**^	−0.32^*^	−0.59^**^
SF36 - 2 -Mental Component Summary	−0.37^**^	−0.23	−0.19	−0.004	−0.25

Pearson correlations between subscales, or the total score of the PRUNE, and the subscales of the SF-36 obtained at follow-up indicate that pain, sensory/motor (SM) symptoms and total scores were consistently moderately correlated with the SF-36 physical subscales or summary score. The sensory/motor symptoms subscale correlated most strongly with overall physical health status. The bolded scores in each column show where the stronger relationships existed. (Correlations who are significant at p<0.05 are noted as *, and those where p<0.01 are noted as **.)

### Factor validity

The baseline factor analysis included the items from the longer version of the PRUNE (before final item reduction). Items dispersed into 4 factors representing pain, symptoms, specific function and usual functions at baseline (Table 8). At baseline, the larger subscales (pain-24% and specific activity-25%) explained the largest portion of the variance. The smaller subset of final items included in the 3 and 24 month factor analysis also loaded on these same 4 factors (See Table 9). Pain explained more than 20% of the variance at 3 and 24 months. Although the item “weakness in the hand” loaded most strongly on its assigned subscale (sensory/motor symptoms function), it exhibited some cross-loading onto Specific Activity. At 24 months recreational activities cross loaded on pain, and specific activity- although its highest loading was on Usual Activity. Overall, factor analysis supported the structural validity of the subscales.

**Table 8:** Factor analysis of the extended version of the baseline PRUNE and contribution to item reduction

**Items on baseline longer version of PRUNE (before final item reduction)**	**Subscale**				**Explanation of final decisions on items deleted from the final scale during production processes**
	**Pain**	**Usual activity /role**	**Specific activity**	**Sensory/motor symptoms**	
Variance Explained by factor (%)	24	15	26	10	Although some items cross loaded as below; the largest amount of variance was by specific activity items; followed by pain, usual activity and sensory/motor symptoms
**Pain - At its worst**	**0.86**	0.12	0.26	0.12	
**Pain - At Rest**	**0.76**	0.21	0.36	0.02	
**Pain - Morning**	**0.74**	0.28	0.33	0.26	
**Pain - During activity**	**0.70**	0.29	**0.51**	0.26	Pain during activity was dropped from the reduced measure
**Pain - After activity**	**0.85**	0.15	0.24	0.19	
**Pain - At night**	**0.83**	0.20	0.25	0.02	
**Pain - Frequency**	**0.89**	0.19	0.11	0.16	
*SM - Numbness*	0.37	*0.74*	−0.02	0.24	Hand weakness cross loads but left as a component of sensory/motor symptoms scale given importance. Motor Control item clarified based on respondent feedback
*SM - Pins And needles*	0.27	*0.76*	0.18	−0.04	
*SM - Control finger*	0.14	*0.78*	0.23	0.13	
*SM - Weakness in hand*	*0.46*	*0.47*	0.06	*0.46*	
SA - Combing hair	0.32	0.25	0.78	0.29	Hair combing deleted: not applicable to a number of respondents
SA - Eating	0.15	0.40	0.67	0.30	
SA - Lift heavy object	0.34	0.18	0.72	0.31	
SA - Finger task	0.49	0.04	0.66	0.37	
**SA - Pull**	0.43	0.12	0.39	0.52	Pulling deleted due to cross loading and correlation with heavy lifting
**SA - Throw a small object**	0.32	0.10	0.81	0.24	Throwing deleted due to higher rate of missingness
SA - Use a telephone	0.39	0.00	0.73	0.26	
**SA - Doing up buttons**	0.12	0.25	0.59	0.21	Doing buttons and washing deleted based on cross correlations and interviews
**SA - Wash opposite armpit**	0.30	0.11	0.73	0.26	
SA - Reaching	0.35	0.01	0.82	0.15	
SA - Turning a doorknob	0.19	0.08	0.69	0.45	
**UA - Personal care**	−0.02	0.06	**0.40**	**0.60**	
**UA - Household**	0.11	0.14	**0.41**	**0.68**	
**UA - Work**	0.14	0.08	0.27	**0.85**	
**UA - Recreational activities**	0.22	0.12	0.37	**0.77**	

Principal Component Analysis with Varimax with Kaiser Normalization for items in the longer version of the PRUNE before the final item reductions were completed. Factor loadings are color-coordinated to highlight loading over 0.40. (Pain subscale items in bold, sensory/motor symptoms items in italics, specific activity items in underline (SA) and usual activity items in bolde (UA). Cross loading occurred on a number of items and was one issue considered in the item reduction process (along with the cross correlation between items, and results of patient interviews with respect to item interpretation and clarity).

**Table 9:** Results of factor analysis of the final PRUNE items at 3 and 24 months after surgery

**Items on Final PRUNE**	**3 months after surgery**				**24 months after surgery**			
	**Pain**	**Usual activity/role**	Specific activity	*SM symptoms*	**Pain**	**Usual activity/role**	Specific activity	*SM symptoms*
Variance explained by factor (%)	**22**	**16**	20	*21*	**30**	**19**	18	*18*
**Pain- worst**	**0.82**	0.09	0.34	0.17	**0.84**	0.25	0.26	0.27
**Pain-at rest**	**0.86**	0.38	0.14	0.23	**0.83**	−0.12	0.32	0.38
**Pain -in morning**	**0.77**	0.38	0.27	0.14	**0.86**	0.09	0.25	0.32
**Pain -after activity**	**0.63**	0.28	0.26	0.16	**0.74**	0.13	0.29	0.27
**Pain- at night**	**0.89**	0.17	0.10	0.23	**0.80**	0.04	0.34	0.33
**Pain- Frequency**	**0.68**	0.14	0.24	0.36	**0.71**	0.21	0.21	0.34
*SM-Numbness*	0.06	0.26	0.19	*0.86*	0.36	0.19	0.15	*0.87*
*SM- Pins and needles*	0.24	0.25	0.10	*0.82*	0.39	0.25	0.20	*0.80*
*SM-Control*	0.36	−0.00	0.17	*0.77*	0.33	0.04	0.23	*0.68*
*SM-Weakness in hand*	0.06	0.15	*0.44*	*0.69*	0.34	0.37	0.48	*0.59*
SA-Eating with fork or spoon	0.30	0.26	0.60	0.10	−0.02	0.88	0.66	0.196
SA- Lift a heavy object	0.23	0.22	0.78	0.14	0.38	0.44	0.58	0.25
SA-Hold an object	0.30	0.47	0.58	0.26	0.37	0.123	0.88	0.14
SA- Finger Task	0.32	0.02	0.78	0.24	0.36	0.38	0.60	0.37
SA-Reaching	0.23	0.22	0.78	0.14	0.45	0.36	0.59	0.31
SA-Turning a doorknob	0.10	0.30	0.70	0.35	0.20	0.30	0.68	0.25
**UA-Personal care**	0.09	**0.84**	0.05	0.34	−0.02	**0.89**	0.27	0.06
**UA-Household**	0.32	**0.76**	0.25	0.35	0.28	**0.52**	0.45	0.11
**UA-Work**	0.35	**0.78**	0.34	0.00	0.32	**0.68**	0.26	0.06
**UA-Recreational activities**	0.36	**0.78**	0.36	0.19	**0.44**	**0.58**	0.48	0.21

Principal Component Analysis with Varimax with Kaiser Normalization. Factor loadings are color-coordinated to highlight loading over 0.40. (Pain subscale items in bold, sensory/motor (SM) symptoms items in italics (SM), specific activity items in underline (SA) and usual activity items in bold (UA). Overall, cross loading was minimal and items differentiated onto the anticipated subscales. Cross loading occurred for item on weakness in grip at both 3- and 24- month follow-up. At 2-year follow-up heavy object and recreational activities also demonstrated some cross-loading. Overall, the loading confirmed the subscale structure.

### Responsiveness

A large effect size (and standardized response mean) was observed from baseline to 24 months (Table 10) for all subscales (all SRM <0.90) and the total score (SRM = 1.55).

**Table 10:** Standardized response means for improvement in PRUNE total and subscale scores from baseline to 24 months

**Subscale**	**Mean change**	**SD**	**Standardized response mean**
Pain	18.8	19.4	0.96
Sensory/motor symptoms	11.8	11.3	1.0
Specific activities	17.0	16.2	1.04
Usual activities	12.9	12.0	0.92
Total score	34.3	19.5	1.55

## Discussion

This study provides evidence that the PRUNE is capable of providing reliable, valid and responsive assessment of symptoms and function experienced by patients with ulnar nerve compression.

Content validity analyses supported the theoretical content of the PRUNE. ICF linking indicated that the PRUNE crosses a number of domains of the ICF. The sensory/motor symptoms scale items linked to ICF codes for touch function, sensation related to the skin, control of movement and muscle power which is a fit with the conceptual target to measure symptoms arising from ulnar nerve compression symptoms. Use of patients, experts, ICF coding and cognitive content coding provided a comprehensive assessment of the content validity of the PRUNE. Given that content validity is the most foundational element of scale validity, it is critical that content issues be rigorously evaluated and resolved before proceeding to more statistically based clinical measurement evaluations. The extent of content validation performed for this study exceeds previous instrument development which is related to the development of clinical measurement methods for ICF linking and use of cognitive interviews in measure development.

Box plots indicated that the full range of scores was endorsed on almost every item. However, there were no participants who reported having “zero” numbness indicating that this was a consistent UNE symptom. Sensory symptoms (numbness and tingling) were rated as being more severe than motor control of the little finger. This is consistent with the pathology of nerve compression where sensory changes are an early impairment and motor changes occur with more severe or prolonged compression [38].

A reliability coefficient of 0.90 has been recommended for measures to be used on individual patients [39]; whereas greater than 0.75 is considered excellent for group comparisons [40]. The reliability of the total score or subscales scores of the PRUNE were all high, indicating either total or component scores could be used to make decisions about individual patients. A minimal detectable change of approximately 7 points for the total score suggests that clinicians should be confident that the PRUNE has indicated a true change in symptoms and disability when the score changes by this amount. Some have suggested that a MDC of less than 10% of the score range is excellent. Whether the ICC, SEM or MDC are used to indicate reliability the PRUNE demonstrated high reliability. We speculate that test-retest reliability can be influenced by the retest interval, the number of items on a subscale, the acuity of the patients tested; and the extent to which the construct being measured is stable and definable by patients. For example, the “usual” activities performed over the past 24 hours can vary and influence how people calibrate that item even when the patient’s condition is stable.

Structural validity was supported by factor analysis that indicated the items fell into 4 subscales that matched the proposed structure. Only minor cross-loading was found. Pain explained more than 20% of the variance at all time points. The confirmation that pain and sensory/motor symptoms systems were separate concepts in the response patterns is important as it verifies the importance of an ulnar nerve specific measure which goes beyond pain questions to capture these additional more disease-specific symptoms.

The known groups validity supported the ability of the PRUNE to discriminate between different clinical subgroups like those who have/have not improved following surgery, or able/not able to return to work. Known group differences can be useful clinically as benchmark comparison scores when assessing whether patient profiles match different categories of outcome. There were 10-fold differences in score between those who rated themselves as asymptomatic versus those who experienced severe symptoms; and a linear pattern was present for the scores for mild, moderate and severe rating. This suggests that increasing PRUNE scores reflect a linear trend of worsening outcomes that mirrors patient and clinician outcome ratings.

The construct validity of the scale was supported since the observed correlations matched the expected convergent relationships. The stronger relationships would be observed between PRUNE subscales and physical subscales of the SF-36 was expected and confirmed. We also anticipated that pain might interfere with social roles which was also confirmed. However, overall, the PRUNE demonstrated low correlation to mental health status which is consistent with its focus on the physical symptoms of UNE.

Finally, the large effect sizes observed in measuring change over time supports the responsiveness of the PRUNE to detect change over time, i.e., following treatment. This is an important measurement feature because assessing change in response to treatment is the most predominant use of outcome measures. A previous study demonstrated a smaller (moderate) effect size for a different PRO, although the intervention was conservative management in that study [14]. Others have cautioned that responsiveness can vary by treatment [35] and thus it would be premature to state that the PRUNE is more responsiveness than this measure.

## Conclusion

This study led to the development of a reliable and valid measurement tool designed specifically for the patient population with ulnar nerve pathology. The next steps in evaluating the PRUNE should include analysis of the measurement properties through Rasch analysis which would address scale and differential item functioning issues (e.g. gender or age effects) not addressed in this study; and analysis of its responsiveness to detect clinical change in head-to-head comparison against other outcome measures. The PRUNE is provided by open access from the developer/copyright owner (J MacDermid- jmacderm@uwo.ca) for free use.

## Competing interests

The authors declare that they have no competing interests.

## Authors’ contributions

JMD developed the Patient-Rated Ulnar Nerve Evaluation, designed the psychometric study, provided oversight to all data collection, conducted the study analyses and wrote the initial drafts of the paper. RG enrolled patients, reviewed data analysis and contributed to revisions of the manuscript. All authors read and approved the final manuscript.

## Pre-publication history

The pre-publication history for this paper can be accessed here:

http://www.biomedcentral.com/1471-2474/14/146/prepub
